# Supplementary material for: Genomic insights into temperature-dependent transcriptional responses of Kosmotoga olearia, a deep-biosphere bacterium that can grow from 20 to 79 °C
Source: Extremophiles. 2017 Sep 11;21(6):963–79. doi: 10.1007/s00792-017-0956-9 (PMC5674127; doi:10.1007/s00792-017-0956-9)
Supplement: Supplementary file 1 — Supplementary material 1 (PDF 164 kb) [file 792_2017_956_MOESM1_ESM.pdf]

# *Supplementary Material*

## Supplementary Methods

### Architecture of the *K. olearia* transcriptome

Transcription start and stop sites were identified using Rockhopper (McClure et al. 2013) on the Illumina transcriptomes (Table S1). The following settings were used: Strand specific, No; Test for differential expression, Yes; Reverse complement reads, No; Orientation of mate-pair reads, forward-reverse; Maximum bases between paired reads, 500 bp; Allowed mismatches, 0.15; Minimum seed length, 0.33; Identify transcript boundaries, Yes; Predict operons, Yes; Minimum expression of UTRs and ncRNAs, 0.5; Minimum reads mapping to a transcript, 20; Minimum transcript length, 50 bp; Minimum count to seed a transcript, 50; and Minimum count to extend a transcript, 5.

## Supplementary Results and Discussion

### The cells respond to the RNA isolation method

Pilot experiments performing RNA-Seq on cultures grown at 65°C not treated with "stop solution" resulted in increased expression of a cold shock-related gene (Kole_2064) (data not shown). We hypothesized that the high expression of a cold shock gene in cultures grown at optimal conditions (65°C) could have been a response to the cell harvesting conditions at 20°C and sought an alternate RNA isolation method. In these transcriptomes we also saw increased expression of a peroxiredoxin gene (Kole_1121), probably a result of these particular cultures being exposed to air during centrifugation in the absence of stop solution.

Therefore, equal volumes of a phenol-ethanol stop solution were added to the cultures prior to harvesting the cells (as described in Materials and Methods). This resulted in much lower expression of the cold shock-related gene (Kole_2064) at 65°C, high expression of expected metabolic genes (e.g., Kole_0379-0382), as well as very high expression of an alcohol dehydrogenase (Kole_0742) (Fig. 3; Table S4). The latter is likely due to exposure of the culture to the phenol-ethanol stop solution at the beginning of RNA extraction, highlighting that a response is provoked by this cell killing/RNA isolation procedure as well. Also, the expression of a redox-sensing transcriptional repressor Rex (Kole_1558) was significantly lower in the 30°C cultures, which suggest that the cultures could have been exposed to some oxygen when injecting stop solution. However, since we saw no increased expression of the peroxiredoxin gene (Kole _1121), we concluded that the effect of oxygen exposure as a result of adding the stop solution is minimal. Thus, since the goal of this study is to elucidate temperature-associated gene expression changes, the cell harvesting method with addition of a phenol-ethanol stop solution was selected for preparation of all subsequent transcriptomes.

**Effect of sequencing platform**

Unexpectedly, Principal Component Analysis (PCA) revealed that the transcriptomes constructed from the same total RNA sample (K65.2 and K65.IT; K40.2 and K40.ML.IT) did not cluster together (Fig. S3A). Instead, within each temperature these transcriptomes have grouped with other samples generated with the same sequencing method (Fig. S3A), suggesting the presence of a platform-specific bias. Given that the Ion Torrent libraries resulted in fewer reads (Table S1), the bias could originate from the lower sequencing depth achieved for the Ion Torrent transcriptomes. In support of this hypothesis, in comparisons of K65-2/K65-IT and K40-2/K40-IT transcriptome pairs the highest RPKM value variability was observed mostly for the low abundance transcripts (data not shown), including those transcripts completely undetected in the IT transcriptome of the analyzed pair (Table

S4). Therefore, to remove the confounding effects of the observed platform-specific bias, all temperature comparisons were performed using only the Illumina-generated transcriptomes, and the Ion Torrent datasets were used only to confirm observed trends.

**Architecture of the *K. olearia* transcriptome**

Rockhopper analysis (McClure et al. 2013) (see Supplementary Methods section) of the Illumina transcriptomes (Table S1) predicted 441 operons that collectively contain 1,716 genes and 475 single gene transcripts (Table S2), including 38 predicted transcripts absent from the current genome annotation (NC_012785.1). Among the 441 operons, 65 were identified as single transcripts with confidently defined boundaries, 288 were predicted to contain multiple transcripts, and the remaining 88 had uncertain boundary predictions. Because a large fraction of the identified operons are predicted to produce multiple transcripts, we estimate a minimum of 475+441=916 transcriptional units (TUs) in *K. olearia*, 52% of which consist of a single gene (Table S2).

**One transcriptome (K65-1) probably entered stationary phase**

In PCA analysis, the K65-1 Illumina transcriptome was positioned between the other Illumina 65°C transcriptomes and the 77°C transcriptomes (Fig. S3A). Inspection of its expression profile revealed that many genes significantly up-regulated at 77°C (Table S5) also showed higher expression in the K65-1 transcriptome, including the extreme heat stress sigma factor-24 (*rpoE*) (Kole_2150), and RNAs *ssrA* (Kole_R0006) and *rnpB* (Kole_R0049) (Table S4, Table S5). On the other hand, other genes involved in heat response, like protease Do (Kole_1599) and the ATP-dependent protease La (Kole_0536), did not show increased expression in K65-1, and most of the genes significantly down-regulated at 77°C did not show decreased expression levels in K65-1, suggesting that the K65-1 culture was not under heat stress. Notably, *rpoE* has been implicated not only in response to temperature stress, but also in a general stress response and in programmed cell lysis in early

stationary phase in *E. coli* (Kabir et al. 2005). Another gene up-regulated in K65-1 (Kole_0545; a ribosome-associated inhibitor, or protein Y) was also shown to be associated with stationary phase, as well as with immediate cold-shock response (Wilson and Nierhaus 2004). Since time of cell harvest was estimated from the growth curves (see Materials and Methods), it can only be considered approximate. Therefore, we suspect that this culture may have entered stationary phase and excluded it from further analyses.

**Predicted sources of laterally transferred genes**

Among 2,118 of *K. olearia*'s protein-coding genes, 354 (17%) were predicted to be acquired by *K. olearia* via lateral gene transfer after the Kosmotogales diverged from other Thermotogae (Table S8; see the main text for discussion of their role in thermoadaptation). Of these, 203 are only present in *Kosmotoga* spp., while the remaining 151 are shared with *Mesotoga* spp. (Table S8). Assigning the putative donor lineage as the top-scoring BLASTP match from the Distal group, we infer that the three largest donor lineages are Firmicutes (181 genes), Archaea (45 genes) and Proteobacteria (29 genes). The predicted gene donors are in concordance with lineages previously identified as involved in gene exchange with Thermotogae (Zhaxybayeva et al. 2012;Nelson et al. 1999;Zhaxybayeva et al. 2009).

**Supplementary References**

Kabir S, Yamashita D, Koyama S, Oshima T, Kurokawa K, Maeda M, Tsunedomi R, Murata M, Wada C, Mori H, Yamada M (2005) Cell lysis directed by $\sigma^E$ in early stationary phase and effect of induction of the rpoE gene on global gene expression in *Escherichia coli*. Microbiology 151: 2721-2735

Kanehisa M, Goto S, Sato Y, Kawashima M, Furumichi M, Tanabe M (2014) Data, information, knowledge and principle: back to metabolism in KEGG. Nucleic Acids Res 42: D199-D205

Katoh K, Misawa K, Kuma K, Miyata T (2002) MAFFT: a novel method for rapid multiple sequence alignment based on fast Fourier transform. Nucleic Acids Res 30: 3059-3066

Katoh K, Standley DM (2013) MAFFT multiple sequence alignment software version 7: Improvements in performance and usability. Mol Biol Evol 30: 772-780

Kolde R (2015) Pretty heatmaps. R package version 1.0.8.

Markowitz VM, Chen IA, Palaniappan K, Chu K, Szeto E, Pillay M, Ratner A, Huang J, Woyke T, Huntemann M, Anderson I, Billis K, Varghese N, Mavromatis K, Pati A, Ivanova NN, Kyrpides NC (2014) IMG 4 version of the integrated microbial genomes comparative analysis system. Nucleic Acids Res 42: D560-7

McClure R, Balasubramanian D, Sun Y, Bobrovskyy M, Sumby P, Genco Ca, Vanderpool CK, Tjaden B (2013) Computational analysis of bacterial RNA-Seq data. Nucleic Acids Res 41: e140

Nelson KE, Clayton RA, Gill SR, Gwinn ML, Dodson RJ, Haft DH, Hickey EK, Peterson JD, Nelson WC, Ketchum KA, McDonald L, Utterback TR, Malek JA, Linher KD, Garrett MM, Stewart AM, Cotton MD, Pratt MS, Phillips CA, Richardson D, Heidelberg J, Sutton GG, Fleischmann RD, Eisen JA, White O, Salzberg SL, Smith HO, Venter JC, Fraser CM (1999) Evidence for lateral gene transfer between Archaea and Bacteria from genome sequence of *Thermotoga maritima*. Nature 399: 323-329

Stamatakis A (2006) RAxML-VI-HPC: Maximum likelihood-based phylogenetic analyses with thousands of taxa and mixed models. Bioinformatics 22: 2688-2690

Wilson DN, Nierhaus KH (2004) The how and Y of cold shock. Nature Structural & Molecular Biology 11: 1026-1028

Zhaxybayeva O, Swithers KS, Foght J, Green AG, Bruce D, Detter C, Han S, Teshima H, Han J, Woyke T, Pitluck S, Nolan M, Ivanova N, Pati A, Land ML, Dlutek M, Doolittle WF, Noll KM, Nesbø CL (2012) Genome sequence of the mesophilic Thermotogales bacterium *Mesotoga prima* MesG1.Ag.4.2 reveals the largest Thermotogales genome to date. Genome Biol Evol 4: 700-708

Zhaxybayeva O, Swithers KS, Lapierre P, Fournier GP, Bickhart DM, DeBoy RT, Nelson KE, Nesbø CL, Doolittle WF, Gogarten JP, Noll KM (2009) On the chimeric nature, thermophilic origin, and phylogenetic placement of the *Thermotogales*. Proc Natl Acad Sci U S A 106: 5865-5870

## Supplementary Figure Legends

**Fig. S1. Growth curves of *Kosmotoga olearia* cultures grown at various temperatures. Panels A through D** compare isothermic growth at a particular temperature to the growth of cultures shifted to that temperature. **Panel E** compares isothermic growth at all tested temperatures. Polynomial regression lines were individually fitted to data from replicate cultures as described in Materials and Methods. Arrows indicate the times at which cultures grown for RNA isolation were harvested. Data points and trend lines are color-coded across all panels according to the growth temperature of the culture used for inoculum (see panel E inset for color assignments).

**Fig. S2. Hierarchical clustering of core energy metabolism genes and genes differentially expressed at different temperatures.** Each gene is represented as a row, and each transcriptome as a column. Genes are labeled using locus tags, and transcriptome label abbreviations are listed in Table S1. Gene clusters were formed using the "average linkage" method, Manhattan distance as a distance metric, and $log_2$-transformed RPKM values as data. Transcriptomes were clustered using “average linkage” method and Euclidean distances. All plots were produced using the *pheatmap* package in R (Kolde 2015). **Panel A**: Clustering of 51 genes potentially involved in energy production during growth on pyruvate (Table S3). Based on consistently high expression values, 16 of these 51 genes are predicted to be part of *K. olearia*'s core pyruvate catabolism, for which gene labels are shown in italic font. Colors represent $log_2$ RPKM values. **Panels B-D:** Clustering of identified temperature-responsive genes at 40ºC (panel B), 30ºC (panel C), and 77ºC (panel D). Colors represent RPKM values scaled across each gene (see color bar to the right of each heatmap; values represent fold change). For each gene the log2RPKM values were centered around 0. Letters next to gene locus tags refer to the COG functional category. COG category abbreviations: *J* translation, ribosomal structure and biogenesis, *K* transcription, *L* replication, recombination and repair, *D* cell cycle control, cell

division, chromosome partitioning, *V* defense mechanisms, *T* signal transduction mechanisms, *M* cell wall/membrane/envelope biogenesis, *U* intracellular tracking, secretion, and vesicular transport, *O* post-translational modication, protein turnover, and chaperones, *C* energy production and conversion, *G* carbohydrate transport and metabolism, *E* amino acid transport and metabolism, *F* nucleotide transport and metabolism, *H* coenzyme transport and metabolism, *I* lipid transport and metabolism, *P* inorganic ion transport and metabolism, *Q* secondary metabolites biosynthesis, transport, and catabolism, *R* general function prediction only, *S* function unknown, NC not in COG database. In addition, locus tags for genes from selected COG categories are color coded as follows: *P* light green, *I* dark green, *C* orange, *G* red, *E* dark blue and *O* light blue. The data used to produce the heatmaps is provided in Table S5. Zoom in to see locus tag labels.

**Fig. S3. Principal component analysis and hierarchical clustering of transcriptomes. Panel A**: Principal component analysis of all transcriptomes. Each transcriptome is denoted by a dot. The dots corresponding to Illumina transcriptomes are outlined by rectangles of the same color. The Ion Torrent generated transcriptomes are outlined by a yellow rectangle. **Panel B**: Hierarchical clustering of all Illumina transcriptomes except K65.1. Each gene is represented as a row, and each transcriptome as a column. Transcriptome name abbreviations are listed in Table S1. Gene clusters were formed using the "average linkage" method, Manhattan distance as a distance metric, and $\log_2$-transformed RPKM values as data. Transcriptomes were clustered using “average linkage” method and Euclidean distances as a distance metric. Colors represent $\log_2$-transformed RPKM values (see color bar to the right of the heatmap). The clustering and plotting was carried out using *pheatmap* package in R (Kolde 2015). The data used to produce the heatmaps is provided in Table S4.

**Fig. S4. Expression of fatty acid synthesis genes at different temperatures.** The fatty acid synthesis genes are arranged along the x-axis. Bars represent average expression levels across all

transcriptomes from a given temperature (except K65-1). Error bars refer to one standard deviation across replicates. Asterisks mark the two genes classified as temperature-responsive: Kole_0968 (significantly differentially expressed in the 40°C vs 65°C comparison) and Kole_0969 (significantly differentially expressed in the 40°C vs 65°C and 30°C vs 65°C comparisons). Fatty acid synthesis genes were identified using KEGG (Kanehisa et al. 2014), as implemented in the IMG portal (Markowitz et al. 2014).

**Fig. S5. Impact of lateral gene transfer on temperature-responsive genes in the *K. olearia* genome.** Proportions of up- and down-regulated genes are shown as positive and negative y-axis values, respectively. Blue, green, and red bars represent proportions at 30°C, 40°C, and 77°C, respectively. P-values were obtained from $\chi^2$-tests, in which the distributions in panels B and C (subsets of all temperature-responsive genes) were compared to that in panel A (all temperature-responsive genes). Note that the proportions were calculated from the total number of temperature-responsive genes in each subset, and since many genes are up- or down-regulated at more than one temperature treatment, the total exceeds 1.

**Fig. S6. Evolutionary histories of three temperature-responsive gene families.** Sequences were aligned using MAFFT v7.309 (Katoh et al. 2002; Katoh and Standley 2013) within Geneious v. 9.1.3. The phylogenetic trees were reconstructed using maximum likelihood method (WAG + Γ substitution model; shape parameter estimated; four rate categories) as implemented in RAxML (Stamatakis 2006) within Geneious v. 9.1.3. Bootstrapping analysis was performed with 100 replicates. Bootstrap support values below 70% are not shown. **Panel A:** Enoyl-(acyl-carrier-protein) reductase II (*fabK*, Kole_0970), a gene involved in fatty acid synthesis. Monophyletic Thermotogae genera are collapsed into triangles and color-coded according to their optimal growth temperature: hyperthermophiles are in red, thermophiles are in orange, and mesophiles are in blue. Two clades

consisting of Firmicutes are also collapsed. **Panel B:** Cold shock proteins (*Csp*) Kole_0109, Kole_1491, and Kole_2064. On this phylogenetic tree, Kole_0109 and its homologs in other *Kosmotoga* spp. cluster outside the Thermotogae clade, suggesting that it was probably laterally acquired. Notably, in the *K. olearia* genome Kole_0109 is adjacent to two genes also predicted to have been laterally acquired (Kole_0110 and Kole_0111, see Table 1 and Table S8). Therefore, although Csp proteins are short and highly conserved, resulting in poor bootstrap support (< 70%) for all the branches of the Csp tree, using the combined evidence we hypothesize that Kole_0109, Kole_0110 and Kole_0111 were likely acquired laterally from an unidentified bacterial lineage in a single transfer event. **Panel C:** PpiC-type PPIases, Kole_1682 and Kole_0383. Only homologs with > 30% amino acid sequence identity to the *K. olearia* homologs were included in the analysis, since the homologs with lower sequence identity could not be confidently aligned.
