# Supplementary figures and images for: Genomic insights into temperature-dependent transcriptional responses of Kosmotoga olearia, a deep-biosphere bacterium that can grow from 20 to 79 °C
Source: Extremophiles. 2017 Sep 11;21(6):963–79. doi: 10.1007/s00792-017-0956-9 (PMC5674127; doi:10.1007/s00792-017-0956-9)

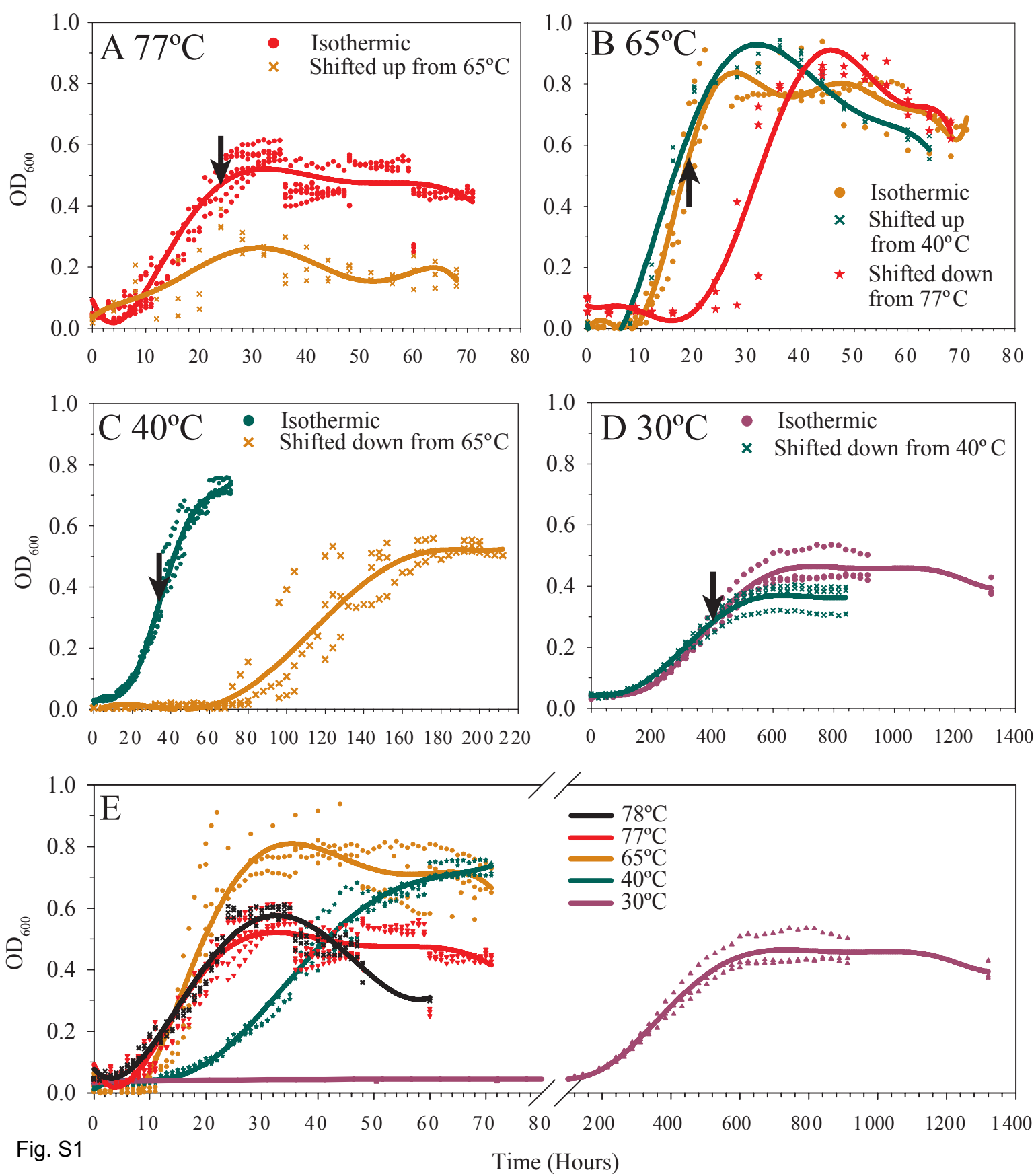

Fig. S1

Fig. S2a

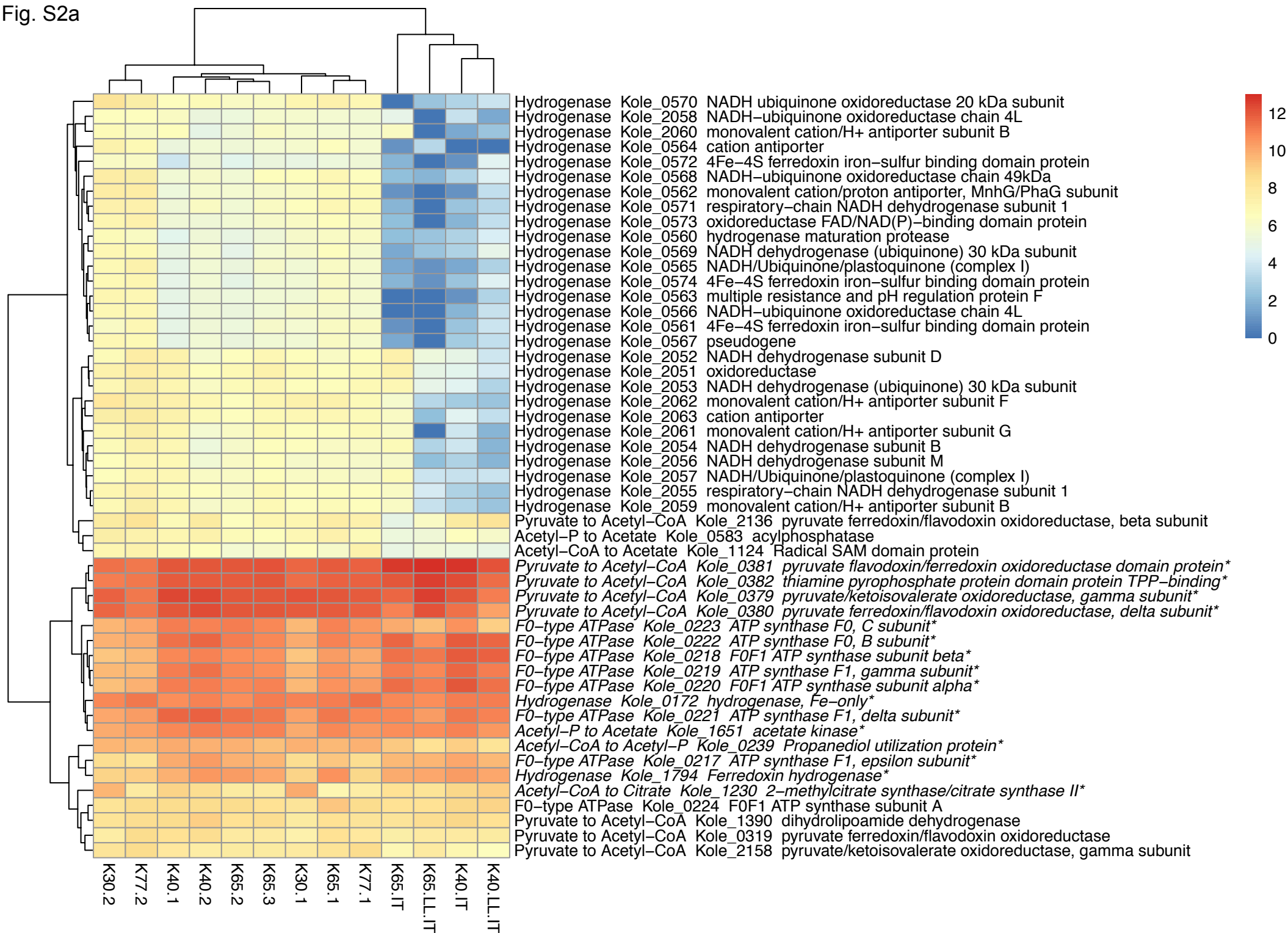

Fig S2b

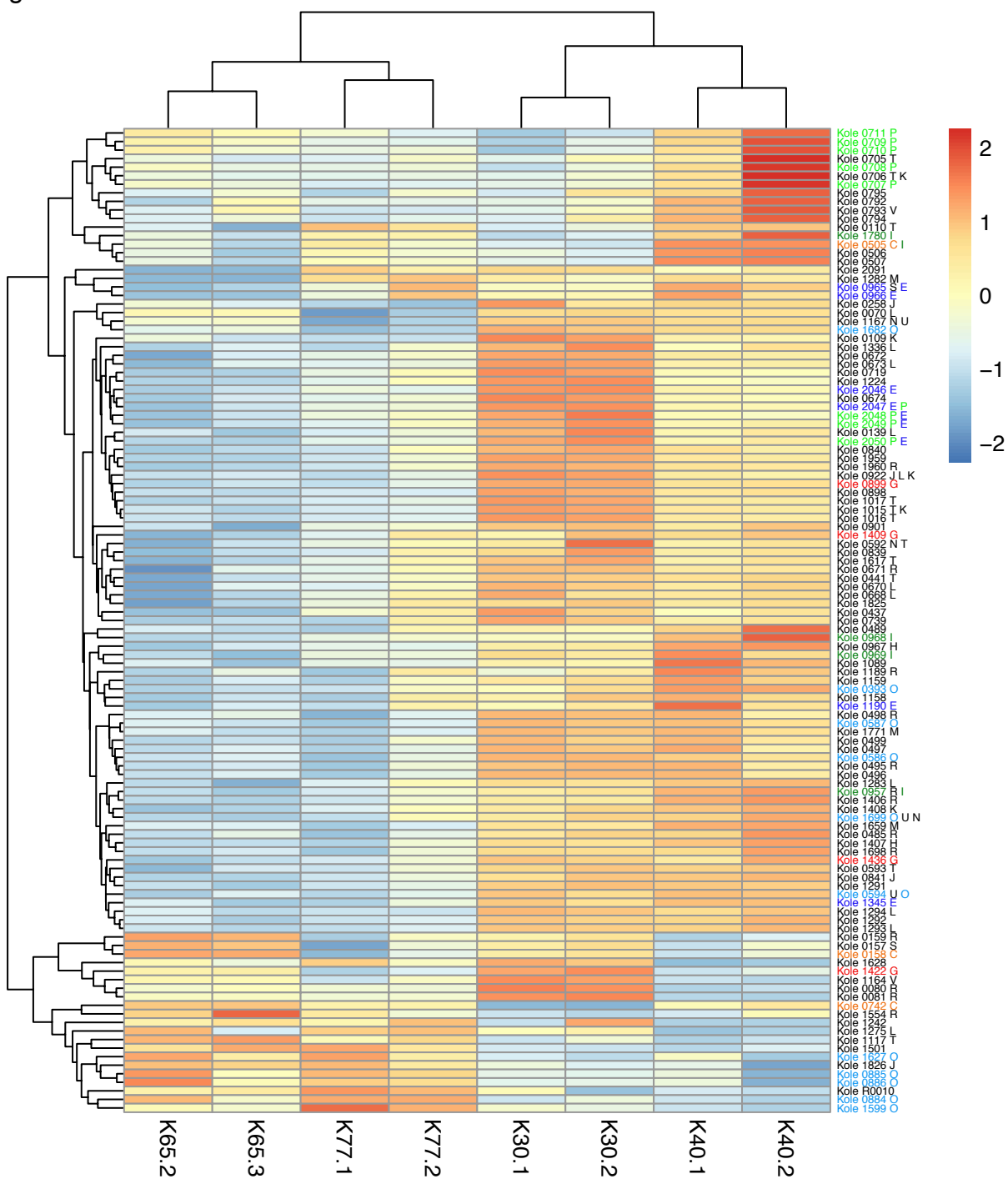

Fig. S2c

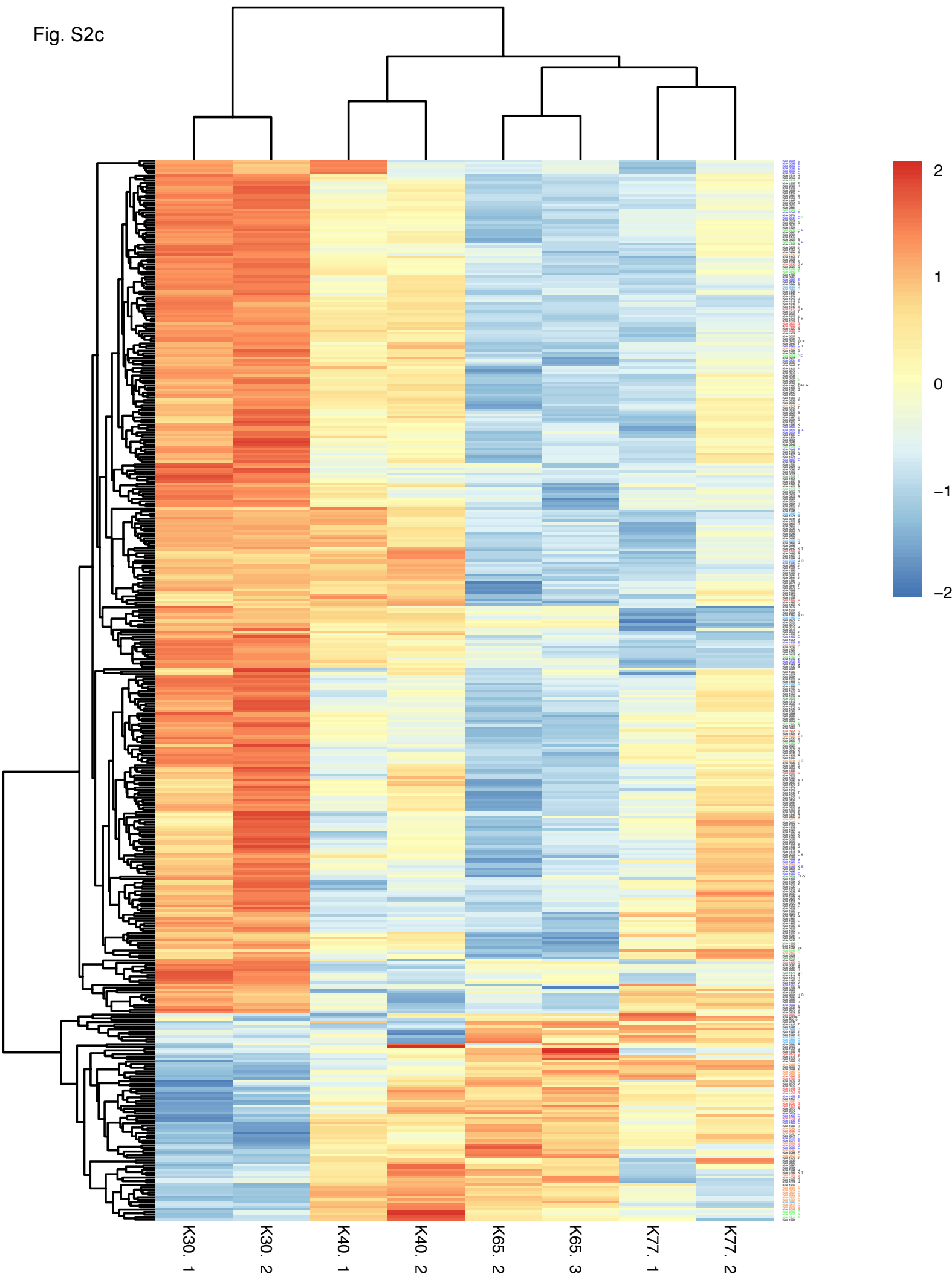

Fig. S2d

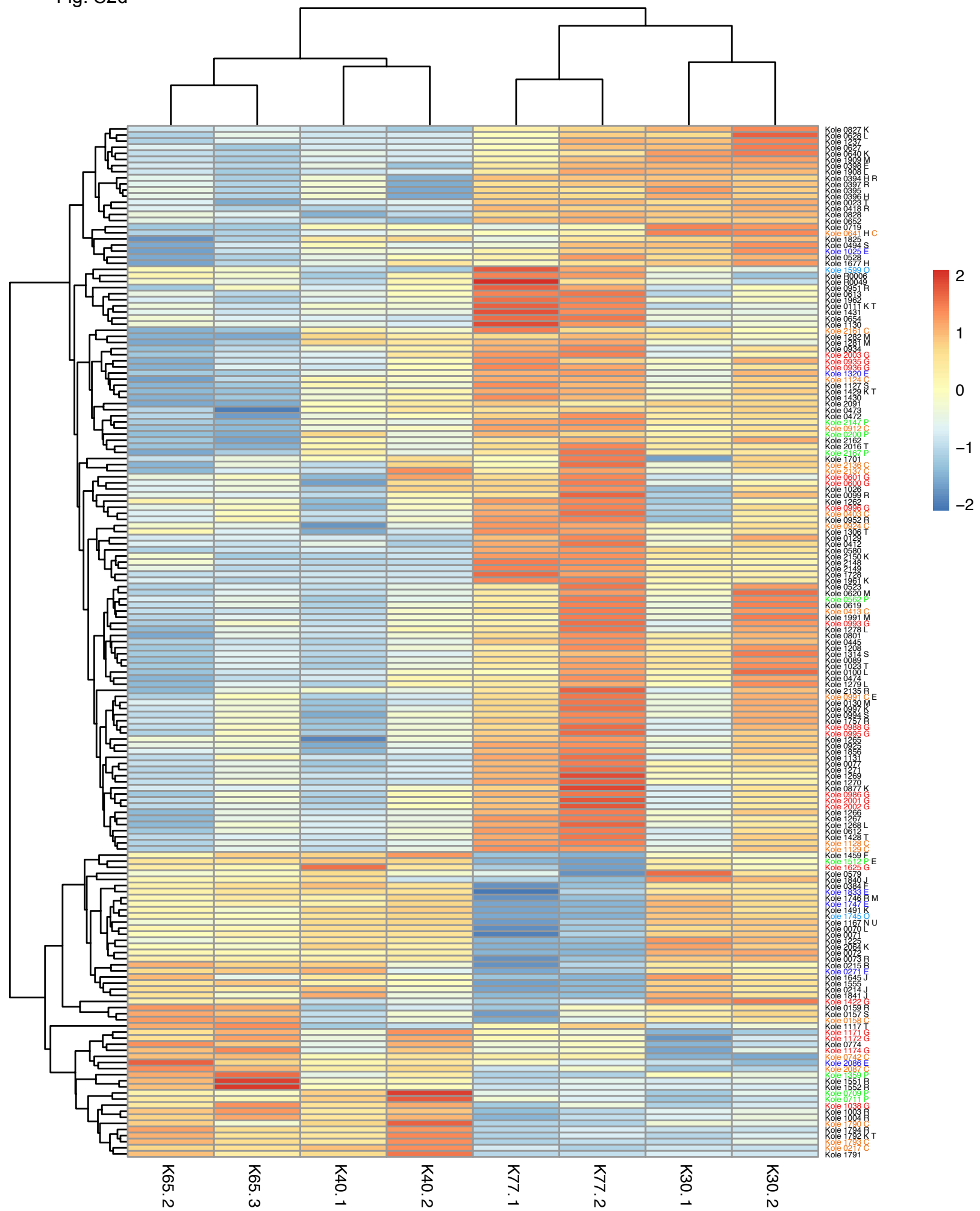

Fig. S3a

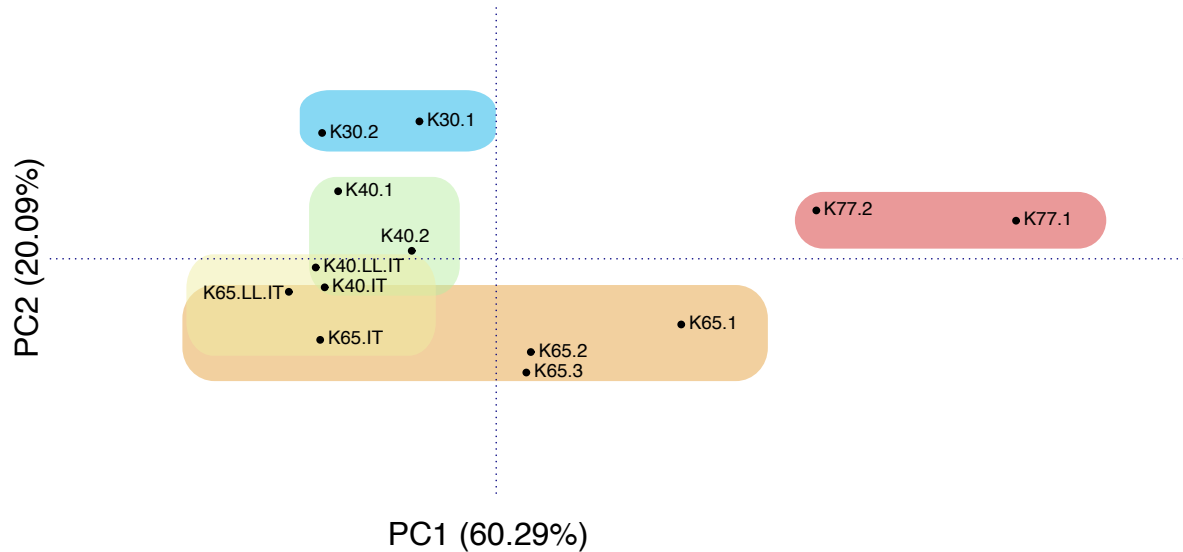

Fig. S3b

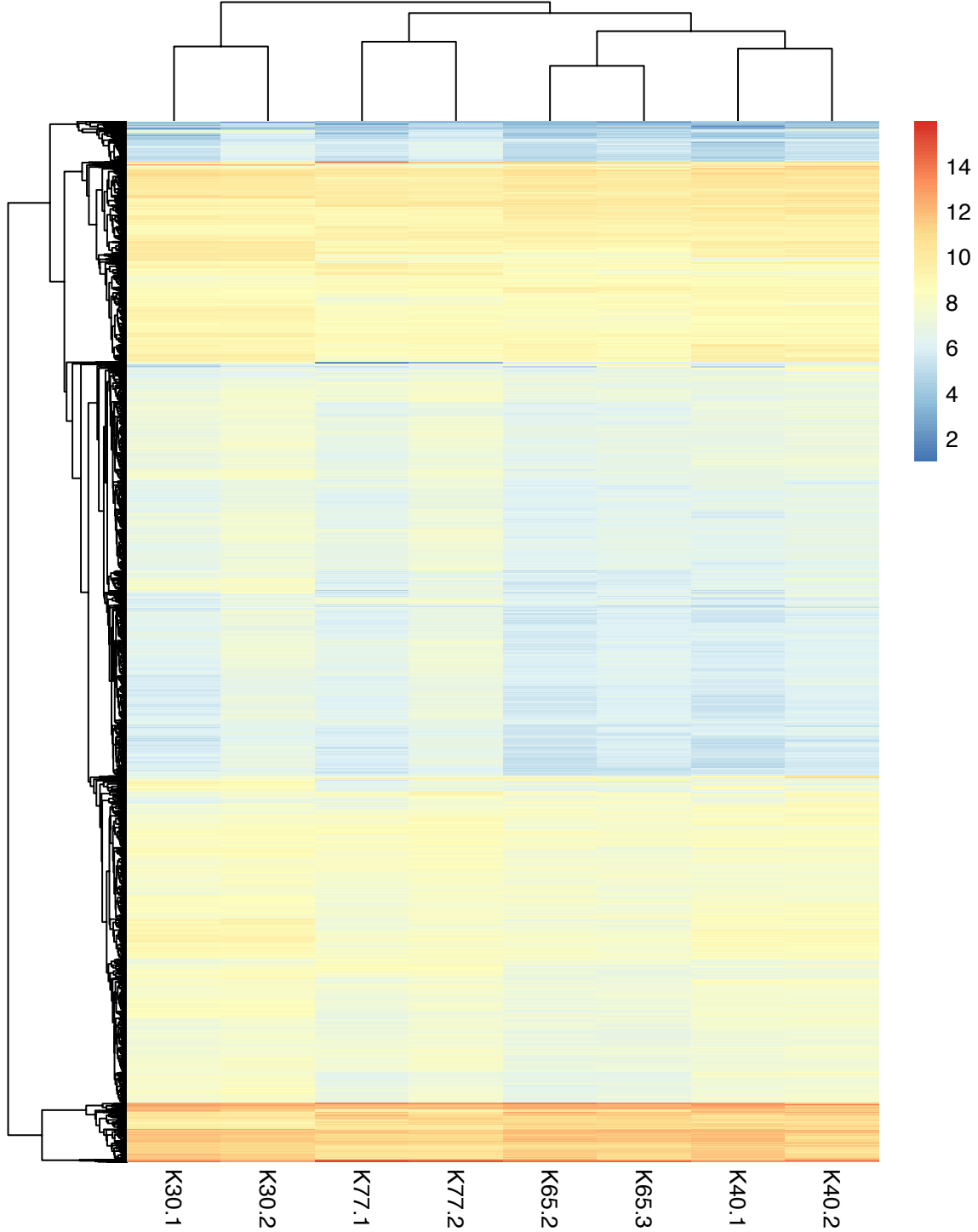

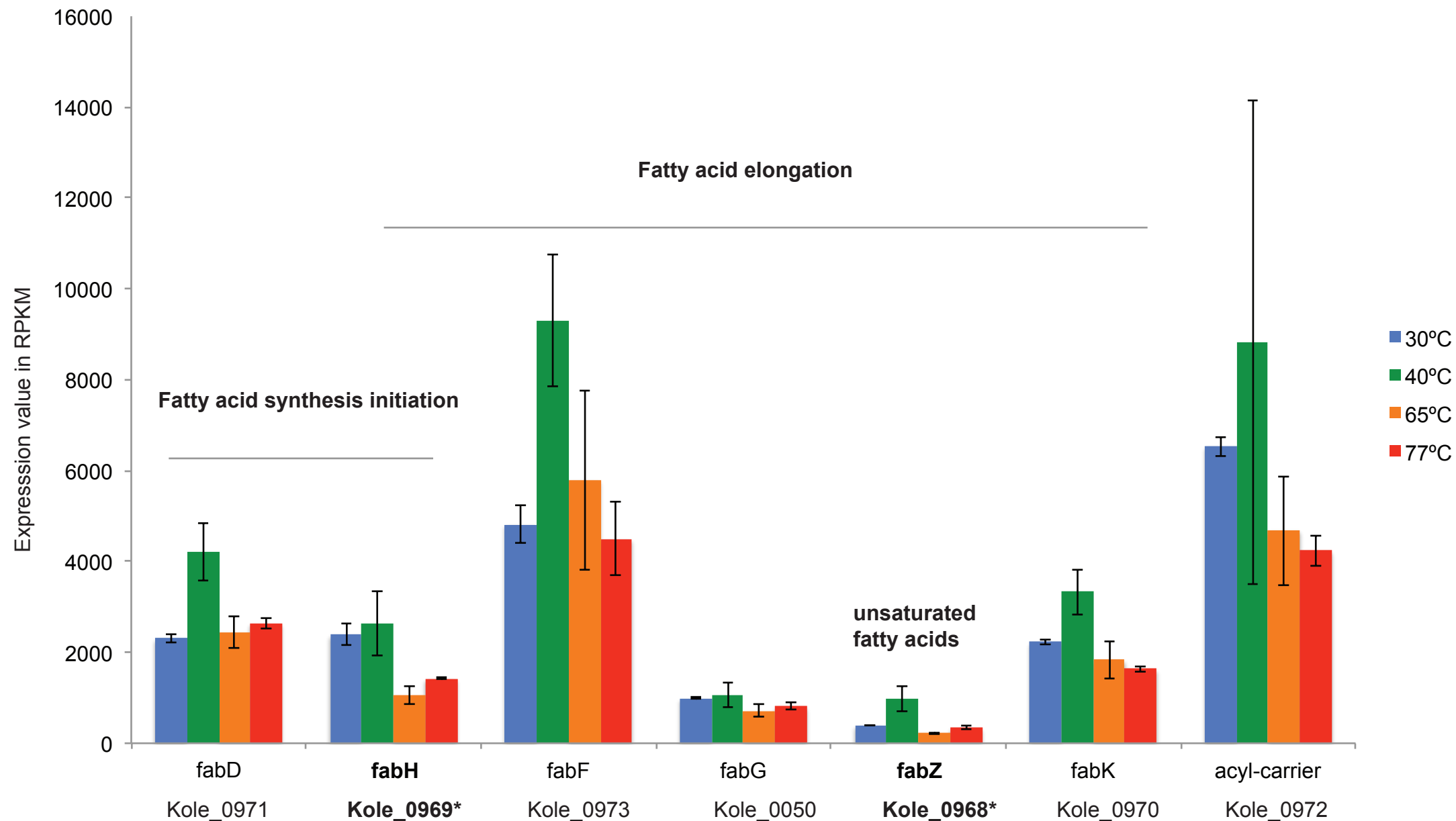

Fig. S4

Fig. S5

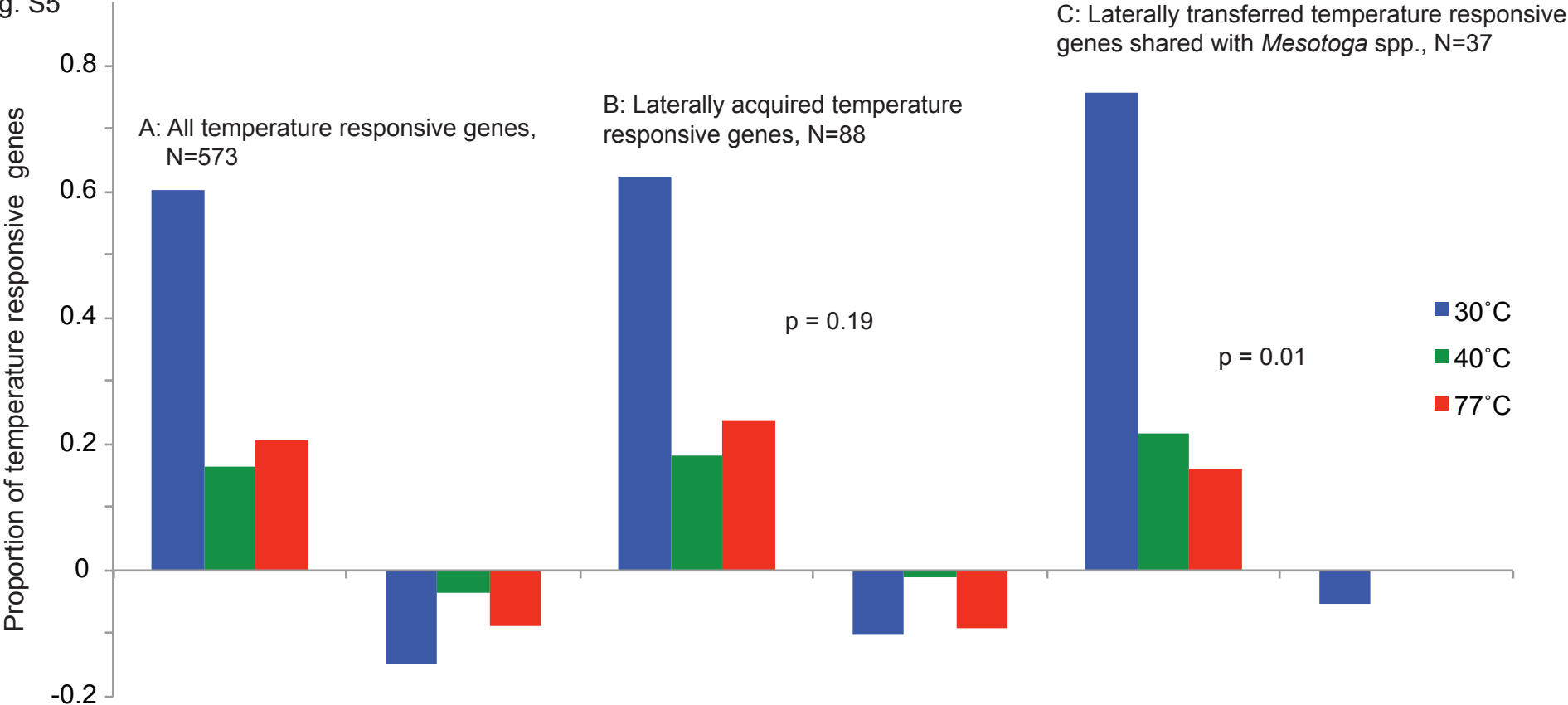

Fig. S6

A

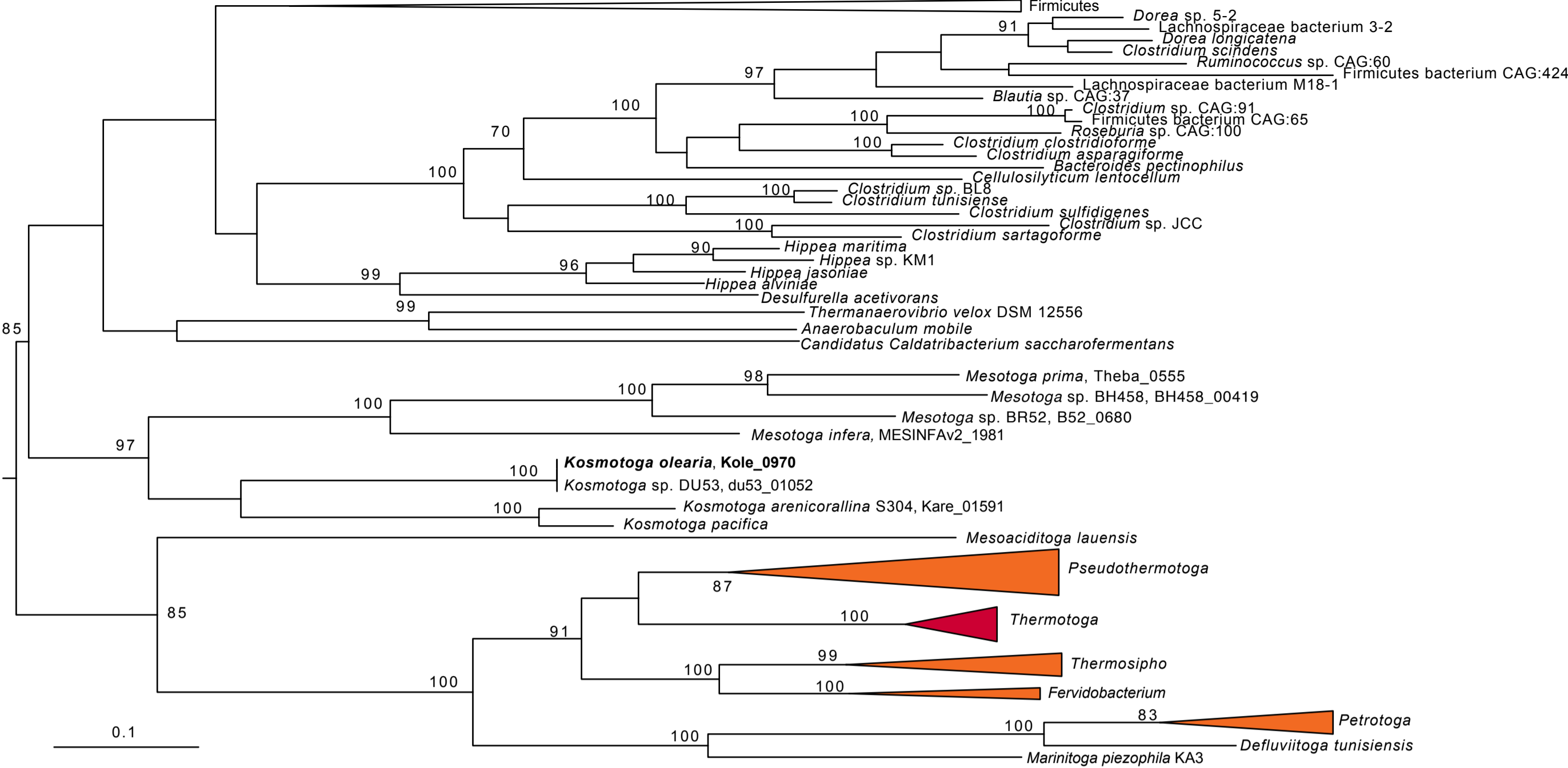

B

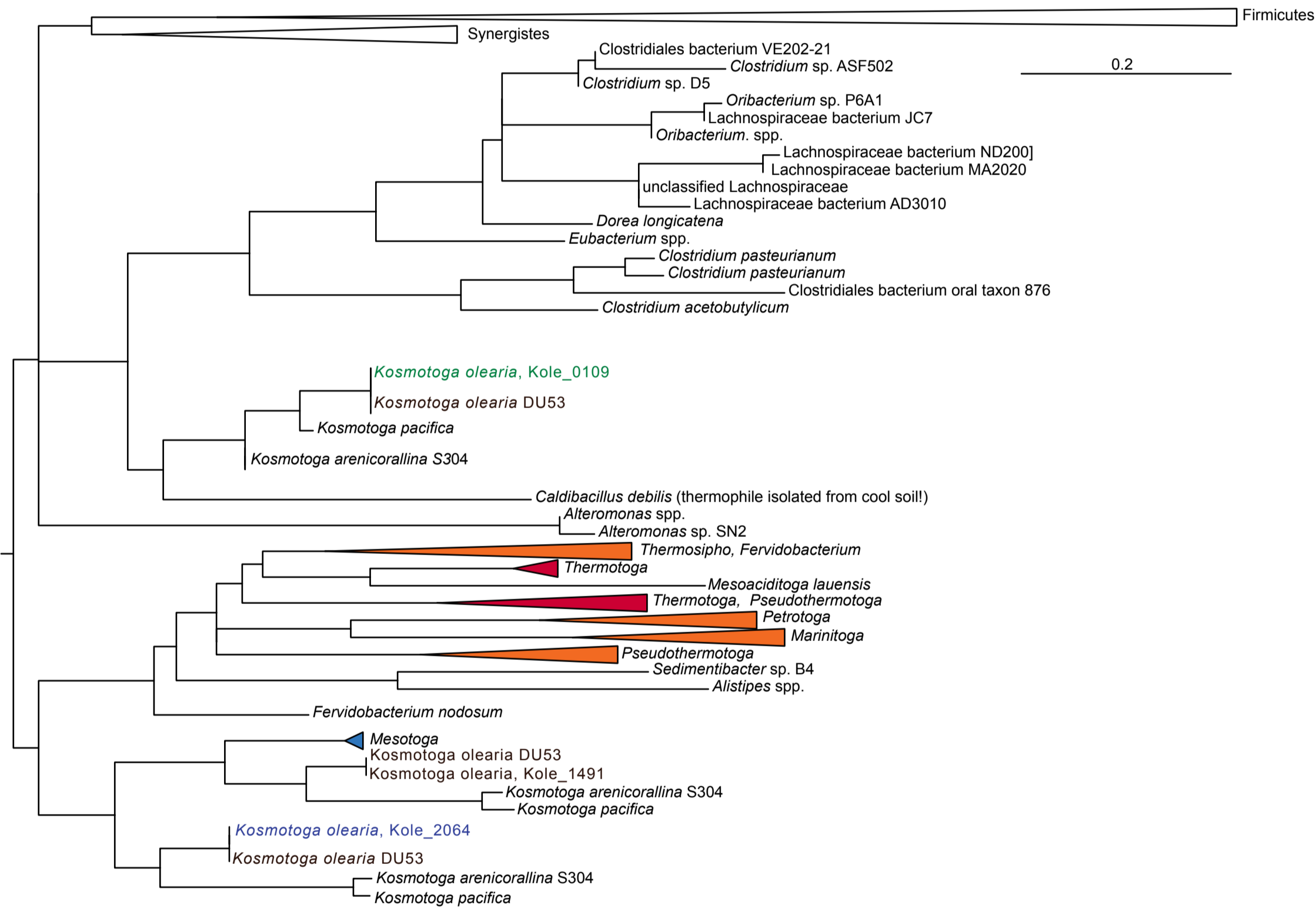

C

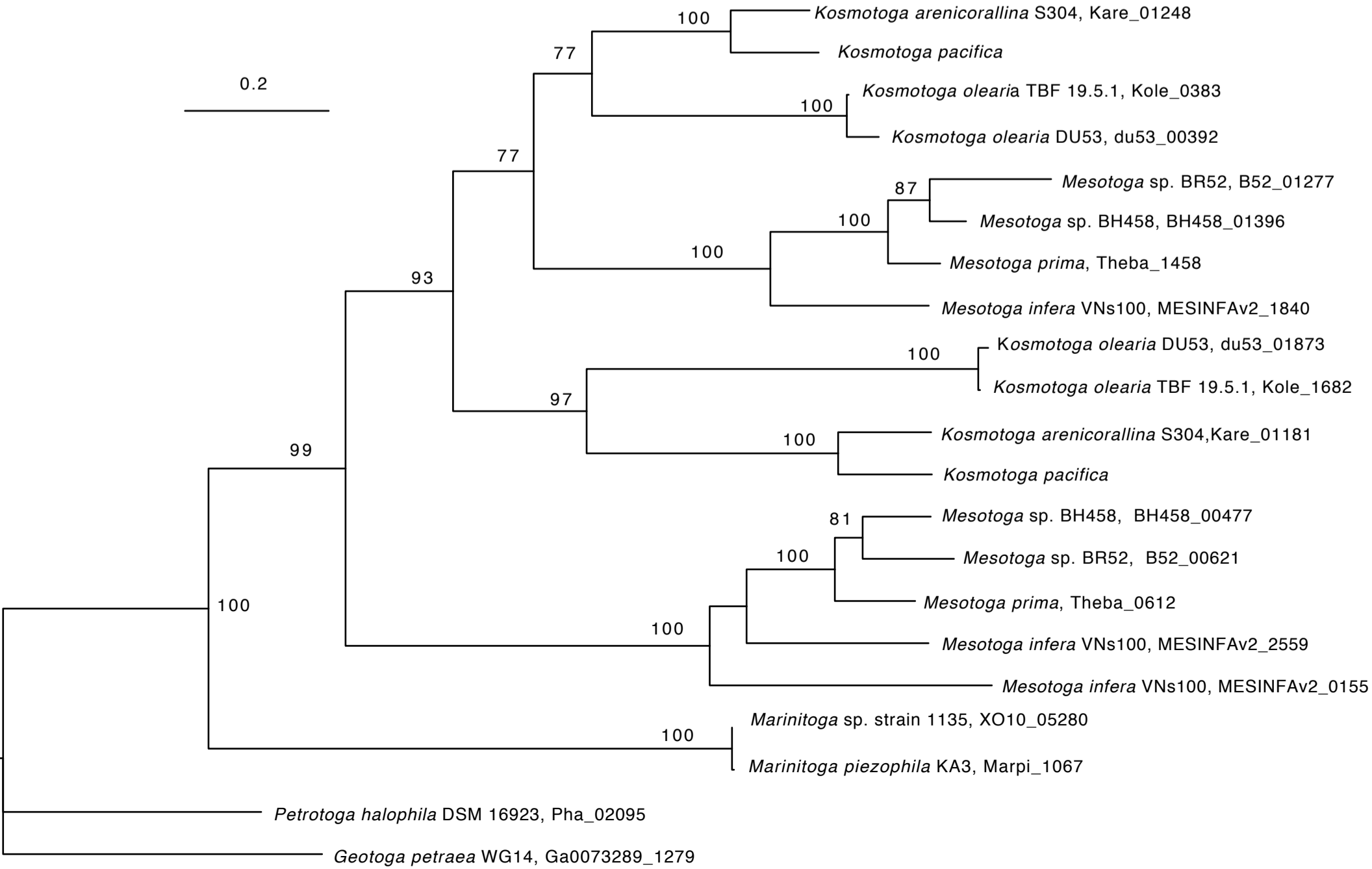

Supplement: Supplementary file 2 — Supplementary material 2 (PDF 921 kb) [file 792_2017_956_MOESM2_ESM.pdf]
